# Will diabetes mellitus induce aplastic anemia by immune factors?: A two-sample and mediation Mendelian randomization study

**DOI:** 10.1097/MD.0000000000044033

**Published:** 2025-08-22

**Authors:** Chuanqi Zhong, Chaoying Duan, Yuan Zeng, Tianhong Guo

**Affiliations:** a Clinical Laboratory, Luzhou Maternal and Child Health Hospital (Luzhou Second People’s Hospital), Luzhou, Sichuan Province, China; b Clinical Laboratory, Deyang Second People’s Hospital, Deyang, Sichuan Province, China; c Clinical Laboratory Diagnostics, The Southwest Medical University, Luzhou, Sichuan Province, China; d Department of Transfusion, The Affiliated Hospital of Southwest Medical University, Luzhou, Sichuan Province, China.

**Keywords:** aplastic anemia, diabetes mellitus, immune cell traits, Mendelian randomization

## Abstract

Diabetes is a prevalent global metabolic and endocrine disorder that is associated with a high incidence of complications and organ system dysfunction. Bone marrow (BM) as a previously neglected site of diabetic end-organ damage, characterized by microangiopathy, neuropathy, fat deposition, and inflammation. As a result, diabetes may lead to negative consequences for physiologic hematopoiesis, which may increase the risk of aplastic anemia (AA). Summary genetic data for diabetes mellitus (DM) were sourced from FinnGen; while the data for AA and immune cell traits were obtained from the IEU Open GWAS database. We performed a two-sample univariable Mendelian randomization (MR) analysis to investigate the causal effects of DM on AA. Simultaneously, multivariable MR was utilized to further estimate the direct effect of subgroup (distinct types) of diabetes on AA. Then, a two-step mediation MR analysis was conducted to examine 731 immune cell traits that may mediate these effects. Several methods were used to evaluate the robustness of the results, including sensitivity analyses with Cochran *Q* statistic, MR-Egger and MR-PRESSO, which also help mitigate potential bias from horizontal pleiotropy. The two-sample univariate MR analysis demonstrated DM was significantly and positively linked to the incidence of AA (IVW, OR = 1.12; 95% CI: 1.05–1.95; *P* = 5.11e−04), which was comparable to the direct effect estimated for type 2 diabetes on AA risk in multivariable MR (multivariable IVW, OR = 1.18; 95% CI: 1.03–1.35; *P* = 1.96e−02). In two-step mediation MR, we explored 54 immune cell traits associated with DM, among these, only the Resting CD4 + regulatory T cell absolute count emerged as a potential mediator influencing the risk of AA, accounting for 10.64% of the effect. We found robust genetic evidence for a causal association between DM and AA risk, and rest CD4+ Treg absolute count, might mediate this effect. However, the potential implications of our findings for AA prevention require validation through well-powered randomized clinical trials.

## 1. Introduction

Aplastic anemia (AA) is a syndrome caused by bone marrow (BM) hematopoietic failure. The main manifestations are pancytopenia in peripheral blood and reduced hyperplasia of BM hematopoietic cells. Clinically, anemia, bleeding and infection are 3 major complications. In recent years, the incidence of AA has been increasing annually, with a notably higher prevalence observed in the Asian population compared to other demographic groups.^[1,2]^ However, the pathogenesis of AA remains unclear. The provocateurs of AA can be drugs, certain diseases of viral etiology in the acute period, metabolic diseases and endocrine pathology, unfavorable working conditions and autoimmune diseases. Several studies have demonstrated immune-mediated etiology plays an important role in the occurrence and development of AA. The dysfunction of T lymphocytes and the increase in hematopoietic negative regulatory factors constitute the pathogenesis of AA.^[3]^

As a global metabolic and endocrine disease with high incidence, diabetes is prone to multiple complications and organ system function damage. Hyperglycemia and metabolic disorders caused by diabetes altered number and function of immune cells, of both innate and acquired immunity.^[4]^ The release of injury-related molecules and the triggering of pro-inflammatory signaling pathways can activate a variety of immune cells and induce multiple organ system complications. For the bone marrow (BM) hematopoietic system, diabetes can enhances adipogenesis in the BM and reduces the number of marrow-resident vascular regenerative stem cells.^[5]^ Moreover, it can reduce the endothelial production of Cxcl12, a quiescence-promoting niche factor that reduces stem cell proliferation, which in turn participate in the dysregulation of BM hematopoiesis.^[6]^ Studies in murine models and humans have identified BM as a previously neglected site of diabetic end-organ damage, characterized by microangiopathy, neuropathy, fat deposition, and inflammation. As a result, diabetes impairs the mobilization of BM stem/progenitor cells with negative consequences for physiologic hematopoiesis, immune regulation, and tissue regeneration.^[7]^ However, the association between diabetes mellitus (DM) and AA is mostly theoretical speculation or sporadic case reports.^[8]^ Whether diabetes can induce AA by inhibiting the hematopoietic function of BM still worth exploring.

Mendelian randomization (MR) is a method based on a large database of genome-wide association studies (GWAS), which uses genetic variation as an instrumental variable (IV) to address causal questions about how modifiable exposures influence different outcomes.^[9]^ In medical research, MR is widely used to investigate the causal relationship between various exposure factors and disease outcomes. It can effectively avoid reverse causality and reduce the influence of confounding factors, thus enhancing the accuracy and robustness of causal inference.^[10]^ Given the low incidence of AA and the challenges associated with controlling diabetes as a confounding factor, conducting observational studies or randomized controlled trials (RCTs) is often impractical. The use of MR for progressive causal inference of diabetes and AA will help to explore the pathogenesis and mutual relationship between the 2 conditions, offering new insights for disease prevention and treatment.

## 2. Date and methods

### 2.1. Study design

**Figure 1.**
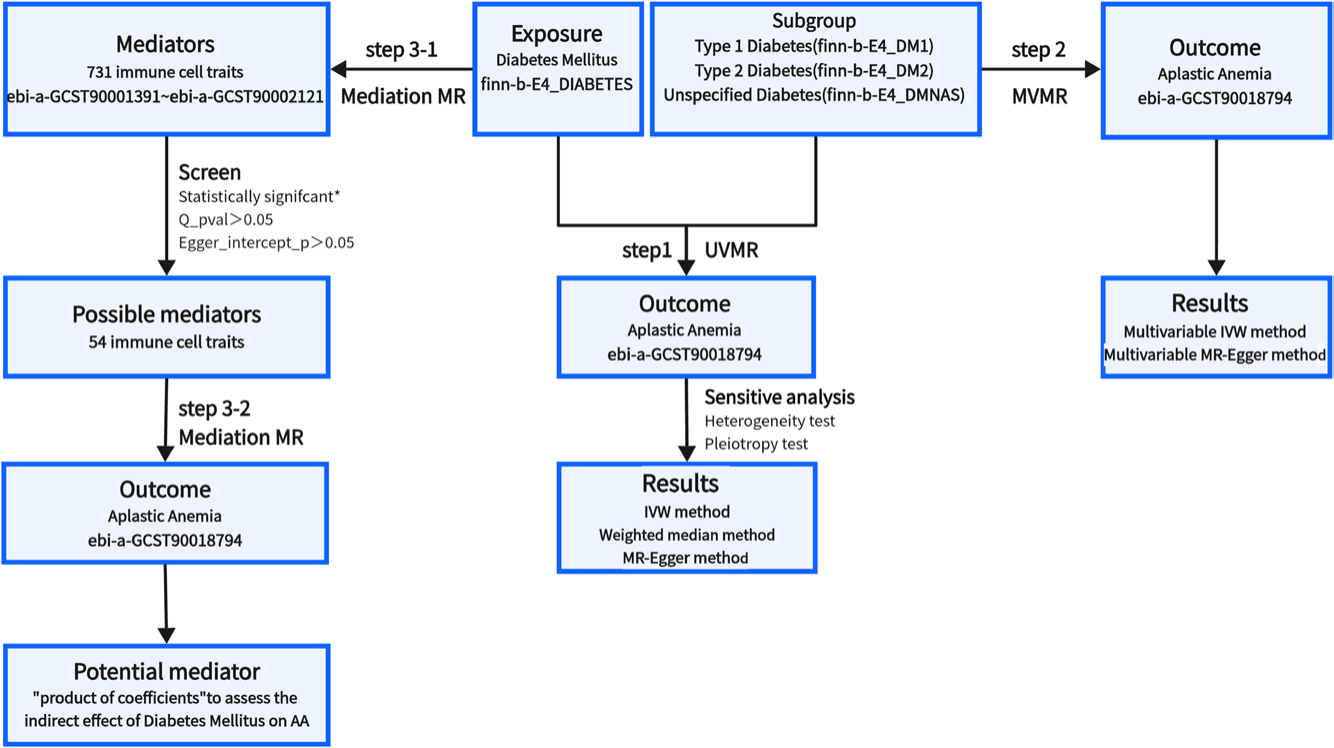
The overview of the study design. *Indicates the results were statistically significant when *P*-value of IVW was <.05 and the direction of weighted median and MR-Egger were consistent with IVW. IVW = inverse-variance weighted.

Step 1: Univariable MR (UVMR) involved analyzing the causal effects of DM (including subgroup of type 1 diabetes, type 2 diabetes, and unspecified diabetes) on AA. Step 2: Multivariable MR (MVMR) was used to further estimate the direct effect of each type of diabetes in the subgroup on AA, independent of the other 2 types of diabetes. Step 3: mediation MR (using a two-step method) consisted of step 3-1, where the causal effect of DM on mediators (731 immune cell traits) was estimated, and step 3-2, which assessed the causal effect of possible mediators on the risk of AA. An overview of the study design is depicted in the diagram (Fig. 1).

We defined single-nucleotide polymorphisms (SNPs) as IVs. MR is based on 3 core assumptions: the IVs are closely associated with the exposure factors; IVs are not associated with confounding factors; IVs do not affect the outcome directly, and it can only affect outcome via the exposure.^[9]^

### 2.2. Data source

The GWAS summary data of DM came from FinnGen biobank (https://www.finngen.fi/en), which has collected and correlated genome and longitudinal health data of more than 500,000 Finns. Data from health registers has been used to define clinical phenotypes (endpoints). The DM (E4_DIABETES) characterized by abnormally high blood sugar levels due to diminished production of insulin or insulin resistance/desensitization has included 3 phenotypes (endpoints), type 1 diabetes (E4_DM1), type 2 diabetes (E4_DM2), and unspecified diabetes (E4_DMNAS).

The genetic data of AA derived from IEU Open GWAS database (https://gwas.mrcieu.ac.uk/). In this cross-population atlas of genetic associations for 220 human phenotypes, researchers curated the past medical history records included in the clinical data, and performed text-mining to retrieve disease records from the free-format electronic medical records as well.^[11]^

For each immune trait, GWAS summary statistics are available in the IEU Open GWAS database (https://gwas.mrcieu.ac.uk/) with accession numbers ranging from GCST90001391 to GCST90002121. The dataset includes a total of 731 immune cell traits, which consist of various features such as absolute cell (AC) counts (118 traits), median fluorescence intensities reflecting surface antigen levels (389 traits), morphological parameters (32 traits), and relative cell counts (192 traits).^[12]^ An overview of each dataset is summarized in Table 1.

**Table 1:** A summary of datasets.

Trait	Phenotype	Data sources	Year	Ancestry	Sample size	GWAS ID
Outcome	Aplastic anemia	IEU OpenGWAS	2021	European	469,372 + 4128	ebi-a-GCST90018794
Exposure	Diabetes mellitus	FinnGen	2021	European	183,185 + 35,607	finn-b-E4_DIABETES
Subgroup of exposure	Type 1 diabetes	FinnGen	2021	European	183,185 + 5928	finn-b-E4_DM1
	Type 2 diabetes	FinnGen	2021	European	183,185 + 32,469	finn-b-E4_DM2
	Unspecified diabetes	FinnGen	2021	European	183,185 + 3580	finn-b-E4_DMNAS
Mediators	731 immune cell traits	IEU OpenGWAS	2020	European	NA	ebi-a-GCST90001391–ebi-a-GCST90002121

GWAS = genome-wide association studies.

Ethical approval for the FinnGen and IEU Open GWAS studies was granted by their respective institutional review boards. All primary studies obtained informed consent from participants. This secondary analysis of de-identified, publicly available summary data was exempt from additional ethical approval per institutional guidelines.

### 2.3. IVs selection

First, we selected SNPs with significant associations for exposure (*P* < 5 × 10^–8^). Then we excluded the SNPs with linkage disequilibrium (LD) in the analysis. The LD of chosen SNPs strongly related to outcome should meet the condition that *R*^2^ < 0.001 and distance > 10,000 kb. Last, to rule out bias due to the insufficient correlation between genetic variation and exposure, which is known as weak instrument bias, we calculated the explained variance (*R*^2^) and *F*-statistic parameters to determine whether the identified IVs were strongly associated with exposure. Generally, SNPs with *F*-statistic parameters < 10 are considered weak instruments.^[13]^ In this study, *R*^2^ = 2 × EAF × (1 − EAF)×β^2^/ [(2 × EAF × (1 − EAF)×β^2^ + 2 × EAF × (1 − EAF) × N × SE^2^)] and *F* = *R*^2^ (N − *k* − 1)/*k*(1 − *R*^2^), where N represents the sample size, *k* means the number of instruments, EAF and SE stand for effect allele frequency and standard error respectively. When extracting the association results between the IVs and the outcome, in case no corresponding SNPs were available in the outcome, we utilized a strongly related SNPs (LD proxy with LD > 0.8) as a substitute. Should the proxy SNP also be undetectable, the SNPs were then excluded from the IVs. The effects of SNPs on exposure and outcome were then harmonized to ensure that the β values were signed to the same alleles. After data harmonization, we removed palindromic SNPs with intermediate allele frequencies (>0.42).

### 2.4. MR analysis

#### 2.4.1. UVMR

To investigate the causal relationship between MD and AA we performed a UVMR analysis. Three most popular MR methods were used: inverse-variance weighted (IVW) test, weighted median and MR-Egger regression. The IVW method is reported to be more powerful than the others under certain conditions.^[14]^ Therefore, we considered the results were statistically significant when *P*-value of IVW was <0.05 and the direction of weighted median and MR-Egger were consistent with IVW.

#### 2.4.2. MVMR

The subgroup (distinct types) of diabetes related to each other, and there were SNPs associated with at least 2 of them. Considering these relationships, we performed a MVMR analysis^[15]^ to simultaneously estimate the direct effect of each type of diabetes on AA, while accounting for the presence of the other 2 types. The *P*-value of IVW multivariable MR <0.05 was considered to be statistically significant. And we used the multivariable MR extension of the MR-Egger method to correct for both measured and unmeasured pleiotropy.^[16]^

#### 2.4.3. Mediation MR

For significant MR associations, a two-step MR analysis was applied to assess mediation. In the first step, genetic instruments for MD were used to estimate the causal effect of the exposure on the mediators (731 immune cell traits). In the second step, genetic instruments for the possible mediators were used to assess the causal effect of the possible mediators on AA risk. For this was an exploratory study, we did not correct for multiple testing in this step.^[17]^ Where there was evidence that MD influenced the mediator, which in turn influenced the AA risk, we utilized the “product of coefficients” method^[18]^ to assess the mediating effect of MD on AA risk via each potential mediator.

### 2.5. Sensitive analysis

To assess the robustness of the results, several sensitivity analyses were performed. First, Cochran Q Statistics was used to test whether the heterogeneity of the results was significant, with *P*-value > 0.05 meant that there was no significant heterogeneity.^[19]^ Then the MR-Egger intercept was utilized to assess the offset due to the IV’s invalidity.^[20]^ Meanwhile, we employed the MR-PRESSO global test to examine the study for any potential horizontal pleiotropy, with a *P*-value > .05 indicating that no significant horizontal pleiotropy exists.^[21]^ Finally, we applied the MR-PRESSO outlier test to determine if any SNP constitutes an outlier. Should an outlier be identified, we eliminated it and reassessed the MR-PRESSO global test.

## 3. Results

### 3.1. UVMR

**Figure 2.**
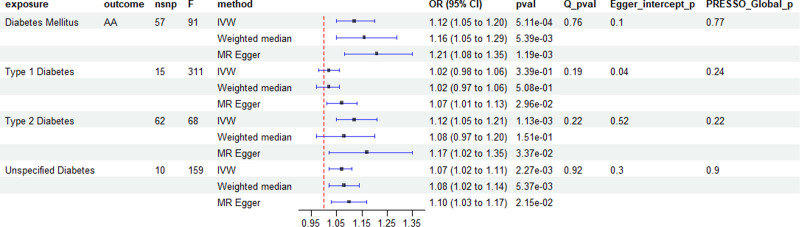
Forest plot of univariable MR analysis of diabetes mellitus on AA. 95% CI = 95% confidence interval, nsnp = number of single-nucleotide polymorphisms, OR = odds ratio, *Q*_pval = *P*-value of Cochran *Q* statistics test.

**Figure 3.**
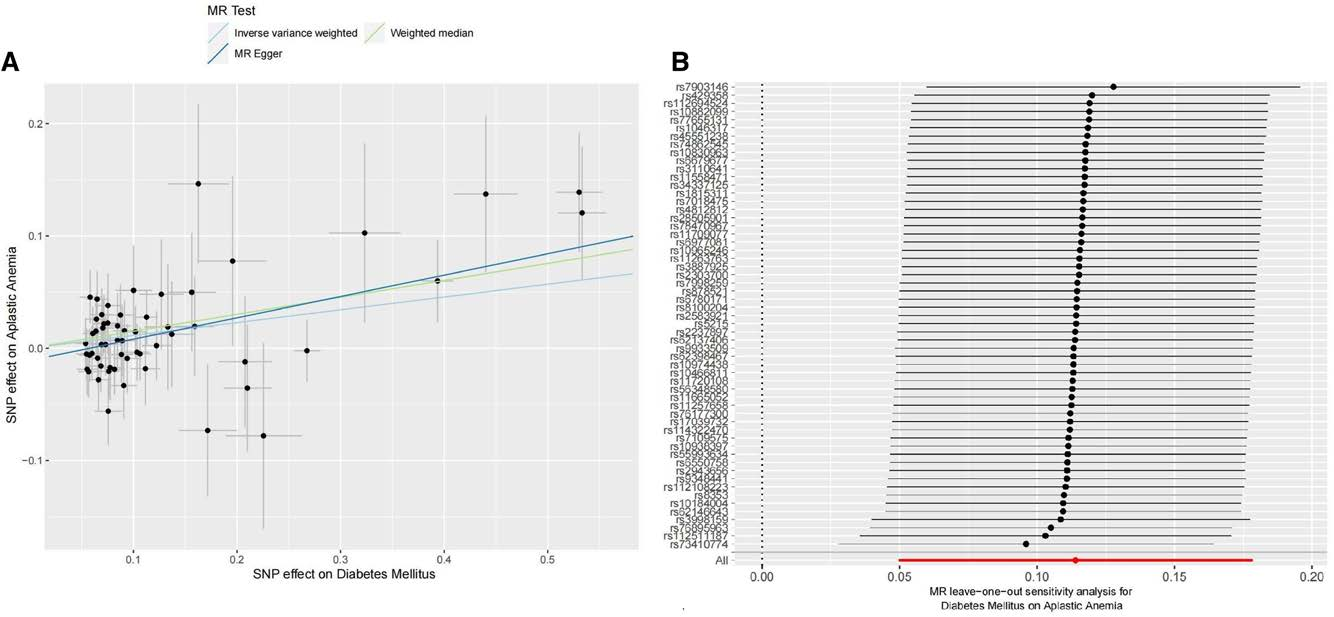
MR plots for the relationship between diabetes mellitus and AA. (A) Scatter plot of SNPs effects on diabetes mellitus versus AA, with the slope of each line corresponding to the estimated MR effect per method; (B) Forest plot shows the individual SNP and outlier of diabetes mellitus on AA in MR leave-one-out sensitivity analysis. Each black line represents the effect of individual SNP on AA, while the gray line represents the effect of outlier, and “All” means the total effect of SNPs on AA. AA = aplastic anemia, MR = Mendelian randomization, SNPs = single-nucleotide polymorphisms.

The number of SNPs extracted from DM and its subgroups, which served as IVs, was illustrated in Figure 2. The F statistics for the genetic instruments were consistent with an absence of weak instrument bias (*F* > 10). The genetic prediction of DM was significantly and positively linked to the incidence of AA, as demonstrated by the two-sample UVMR analysis (IVW method, OR = 1.12; 95% CI: 1.05–1.95; *P* = 5.11e−04). The estimates were similar in size in weighted median (OR = 1.16; 95% CI: 1.05–1.29; *P* = 3.29e−03) and MR-Egger (OR = 1.21; 95% CI: 1.08–1.35; *P* = 1.19e−03). Scatter plots of the DM and AA risk association for the instruments are presented in Figure 3A, with colored lines representing the slopes of the different MR methods. Simultaneously, subgroup analysis indicated, except for type 1 diabetes (IVW method, *P* = .35) which didn’t shown significant link to AA risk, both type 2 diabetes and unspecified diabetes had significant effect on AA risk through two-sample univariate MR analysis (IVW method, OR = 1.12; 95% CI: 1.05–1.21; *P* = 1.13e−03/OR = 1.07; 95% CI: 1.02–1.11; *P* = 2.27e−03, separately).

The results of sensitive analysis are shown in Figure 2. As we can see, All the analysis with a *P*>0.05 in Cochran Q statistic test and MR-PRESSO global test, indicating the results did not exist any significant heterogeneity and there was no potential horizontal pleiotropy across instrument effect. In the MR-PRESSO outlier test we didn’t detect any potential outliers. The forest plot (Fig. 3B) has shown the individual SNP and outlier of DM on AA in MR leave-one-out sensitivity analysis. Additionally, MR-Egger intercept analysis found no evidence of directional pleiotropy except for Type 1 Diabetes on AA (Egger intercept *P* = .04).

### 3.2. MVMR

The independent instruments extracted for multivariable MR are listed in Table S1 (Supplemental Digital Content, https://links.lww.com/MD/P725). The direct effect estimated for Type 2 Diabetes on AA was comparable to the univariable IVW estimate (univariable IVW, OR = 1.12; 95% CI: 1.05–1.21; *P* = 1.13e−03. Multivariable IVW, OR = 1.18; 95% CI: 1.03–1.35; *P* = 1.96e−02). The CIs of multivariable MR-Egger were wider than those of multivariable IVW (multivariable MR-Egger, OR = 1.26; 95% CI: 1.08–1.47; *P* = 2.76e−03) (Table 2). The multivariable MR-Egger intercept analysis did not provide conclusive evidence for horizontal pleiotropy (*P* = .057). The multivariable MR estimates for Type 1 Diabetes (Multivariable IVW, OR = 1.13; 95% CI,0.96–1.32; *P* = .07) and Unspecified Diabetes (Multivariable IVW, OR = 0.81; 95% CI: 0.63–1.06; *P* = .12) were not significant (Table 2).

**Table 2:** Direct effect of each type of diabetes on AA estimated by multivariable MR.

Methods	Subgroup	Numbers of SNPs	OR (95% CI)	*P*
Multivariable IVW method	Type 1 diabetes on AA	67	1.13 (0.96–1.32)	1.33e−01
	Type 2 diabetes on AA		1.18 (1.03–1.35)	1.96e−02*
	Unspecified diabetes on AA		0.81 (0.63–1.06)	1.24e−01
Multivariable MR-Egger method	Type 1 diabetes on AA	67	1.16 (0.99–1.36)	6.55e−02
	Type 2 diabetes on AA		1.26 (1.08–1.47)	2.76e−03*
	Unspecified diabetes on AA		0.79 (0.61–1.03)	8.19e−02
	Intercept		–	5.66e−02

AA = aplastic anemia, CI = confidence interval, MR = Mendelian randomization, OR = odd ratio, SNPs = single-nucleotide polymorphisms.

* The *P*-value of IVW < .05.

### 3.3. Mediation MR

**Figure 4.**
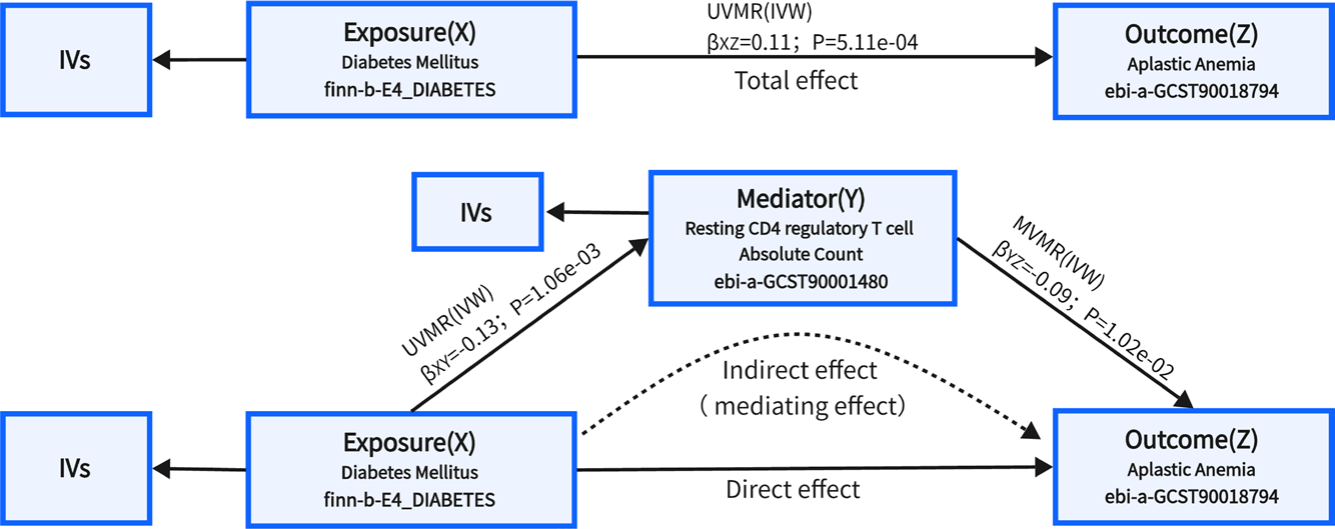
Mediation MR analysis of diabetes mellitus on AA via resting CD4 regulatory T cell absolute count. Total effect = direct effect + indirect effect (mediating effect); the effect of exposure on mediator (β_*XY*_) was estimated by univariable MR, and the effect of mediator on outcome (β_*YZ*_) was estimated by multivariable MR; mediating effect = β_*XY*_ × β_*YZ*_ (coefficient multiplication methods). AA = aplastic anemia, MR = Mendelian randomization.

To identify immune cell traits that could be potential mediators, in the first step, we explored the effect of DM on the 731 immune cell traits. Ultimately, we screened 54 immune cell traits (possible mediators) associated with DM, including 8 traits of AC counts, 32 traits of median fluorescence intensities reflecting surface antigen levels, 2 traits of morphological parameters, and 12 traits of relative cell counts. The results of 3 methods and sensitive analysis are presented in Table S2 (Supplemental Digital Content, https://links.lww.com/MD/P725). In the second step, we assessed the causal effect of the possible mediators on AA risk using genetic instruments for the 54 immune cell traits derived above. After screening, only one possible mediated immune cell trait was obtained, the Resting CD4 regulatory T cell Absolute Count (rCD4 + Treg AC) (IVW method, β = −0.09; 95% CI: −0.17 to −0.005; *P* = 3.65e−02). We then explored the mediating effect of the rCD4 + Treg AC in the impact of DM on AA. As shown in Figure 4, rCD4 + Treg AC, as a potential mediator, significantly increased the risk of AA induced by DM (mediating effect: 10.64%, 95% CI: 0.94%–20.33%, *P* = 3.15e−02) (Table 3).

**Table 3:** The mediating proportion of diabetes mellitus on AA via rCD4 + Treg AC.

Mediator	Total effect β_*XZ*_ (95% CI)	Direct effect β_*XY*_ (95% CI)	Direct effect β_*YZ*_ (95% CI)	Mediated proportion (%)* (95% CI)	*P*
rCD4 + Treg AC	0.11 (0.05–0.18)	−0.13 (−0.21 to −0.05)	−0.09 (−0.15 to −0.02)	10.64 (0.94–20.33)	3.15e−02

AA = aplastic anemia, CI = confidence interval, rCD4+ Treg AC = resting CD4 regulatory T cell absolute count.

* Mediated proportion (%) = direct effect β_*XY*_ × direct effect β_*YZ*_/total effect β_*XZ*_.

## 4. Discussion

In this study, by using genetic variants as unconfounded proxies for DM, we observed evidence indicating that diabetes may be the predisposing factor of AA. We used several methods to infer robust causal estimates, including sensitivity analyses with Cochran Q statistic, MR-PRESSO, weighted median and MR-Egger, which also prevented the horizontal pleiotropy from conducting bias of effect estimation. Meanwhile, multivariate MR analysis was performed for different types of diabetes in subgroup, and it was found that Type 2 Diabetes was still significantly associated with the risk of AA after considering the influence of Type 1 Diabetes and Unspecified Diabetes. We also conducted a mediation analysis to estimate potential mediators for 731 immune cell traits and showed that the effect of diabetes on AA risk was partially mediated by rCD4 + Treg AC, although the mediated proportion was not remarkably high.

The mechanism by which diabetes induces AA may involve the direct toxic effects of hyperglycemia, microangiopathy, and metabolic abnormalities that interfere with hematopoiesis. The accumulation of advanced glycation end products (AGEs) and the excessive production of reactive oxygen species may damage the stromal cells and vascular endothelial cells in the BM microenvironment, disrupt the survival support for hematopoietic stem cells (HSCs), and lead to DNA damage and mitochondrial dysfunction in HSCs, inhibiting their proliferation and differentiation abilities.^[22]^ Abnormalities in the insulin signaling pathway may affect the energy metabolism of HSCs (such as glycolysis and mitochondrial function), inhibiting their proliferation.^[23,24]^ In addition, diabetes can also activate the autoimmune response, induce an imbalance of pro-inflammatory factors, and promote the abnormal activation of T cells, that directly or indirectly inhibiting the proliferation of HSCs or inducing their apoptosis.^[4,25,26]^ Since Type 1 Diabetes, Type 2 Diabetes, and Unspecified Diabetes are interrelated yet distinct in terms of pathogenesis, age of onset, clinical symptoms, treatment methods, genetic factors, and the risk of complications. Therefore, conducting subgroup and multi-factor analyses of them can help to discover and understand the impact of diabetes as an exposure factor on the risk of AA more comprehensively. In our multivariable MR analysis, even after taking into account the effects of Type 1 Diabetes and unspecified diabetes, Type 2 Diabetes still showed a significant causal relationship with the risk of AA (Multivariable IVW OR = 1.18; 95% CI: 1.03–1.35; *P* = 1.96e−02), which indicated that Type 2 Diabetes may be an independent risk factor for inducing AA compared with other types of diabetes.

Naturally occurring CD4 + regulatory T cells (Tregs), which specifically express the transcription factor FoxP3 in the nucleus and CD25 and CTLA−4 on the cell surface, are a functionally distinct T cell subpopulation actively engaged in the maintenance of immunological self-tolerance and homeostasis.^[27]^ Emerging data suggest, in diabetes patients, function of specific T-lymphocyte populations, including Tregs has altered.^[4]^ In the peripheral blood of diabetic patients, the level of CD4 + Tregs is significantly disordered. The level of CD4 + Tregs in patients with Type 1 Diabetes decreases significantly.^[28]^ On the other hand, the level of CD4 + CD25 + FoxP3 + Tregs in patients with Type 2 Diabetes is closely related to the disease progression, poor blood glucose control, and the risk of complications.^[29]^ In the first step of Mediation MR, univariable MR identified a causal relationship between DM and CD4 + Tregs, i.e., diabetes was associated with a decrease of rCD4 + Treg AC (IVW method, β = −0.13; 95% CI: −0.21 to −0.05; *P* = 1.07e−03). Currently, there are several MR studies that have explored the causal relationship between immune cell traits and diabetes. For example, Li et al demonstrated that higher circulating monocytes and T-lymphocyte subpopulations predicted an increased susceptibility to Type 2 Diabetes.^[30]^ However, there have been no studies that take immune cell traits as an outcome or mediator to explore how diabetes acts on immune cells and thus affects other diseases.

In the second step of mediation MR, we found decrease of rCD4 + Treg AC was associated with increase of AA risk in the consideration of MD (Multivariable IVW method, β = −0.09; 95% CI: −0.15 to −0.02;*P* = 1.02e−02), which confirmed the previous findings. Shi et al found defective immunosuppression by Tregs could contribute to impaired hematopoiesis conducted by effector T cells in vitro, this provided powerful evidence that impairment of Tregs played a critical role in the pathophysiology of AA.^[31]^ Kordasti et al found activated and resting Tregs were reduced in AA.^[32]^ Numerous studies in recent years have also shown that patients with AA indeed have an abnormal distribution of T cell subsets, a decrease in the number of Tregs and an imbalance in the ratio between CD4 + Tregs and Th17 cells.^[33]^ However, the MR Study in 2024 demonstrated that CD39 + Treg cells (CD39 + resting Treg % CD4 Treg and CD39 + secreting Treg AC) were harmful to AA, OR of CD39 + resting Treg % CD4 Treg on AA risk was estimated to be 1.034 (95% CI: 1.010–1.059, *P* = .005), OR of CD39 + secreting Treg AC on AA risk was estimated to be 1.050 (95% CI: 1.013–1.089, *P* = .007), which means the highly active CD4 + Treg may probably increase the risk of AA.^[34]^ This is inconsistent with our results and the possible reasons may be as follows. Firstly, in the method of selection of IVs, we set a more strict significance level (*P* < 5 × 10^–8^) than previous studies (*P* < 1 × 10^–5^) to extract IVs for each immune cell trait, which enhanced reliability of causal inference, improved quality of IVs and make greater resistance to pleiotropy and confounding in our study. Secondly, immune signatures in different states may play a different role in pathophysiology of AA. The expression of CD39 in Tregs may facilitate its involvement in other critical immunosuppressive functions. Therefore, further research is necessary to elucidate the intricate relationship between T cells and the risk of AA.

This two-sample mediation MR study investigating the association between genetic liability for DM and AA risk possessed several strengths and limitations. We used summary genetic associations from the largest and newest available GWAS, allowing us to include the largest possible number of instruments for the exposures, resulting in increased statistical power. At the meantime, the samples of the exposure could be divided into 3 major types, made it possible for us to compare the genetic influence from distinct types of diabetes on AA risk. Moreover, this was the first time to identified rCD4 + Treg AC as a potential mediator between diabetes and AA risk. Admittedly, the results of this study should be carefully interpreted in conjunction with its limitations and those of MR in general. First, despite selecting strongly associated SNPs (*P* < 5 × 10^–8^), the genetic variants explained a small fraction of the total variance in diabetes and cannot be considered exact proxies of the exposure.^[35]^ Second, we didn’t use multiple-testing correction to adjust the *P*-value of IVW when extracting genetic instruments for 54 immune cell traits to assess the causal effect of the possible mediators on AA risk. This may lead to an increased false positive rate of potential mediators, even if we used a series of sensitivity tests to reduce weak IVs and horizontal pleiotropy. Third, although MR provides an important alternative for verifying effects, the association between DM and AA is mostly theoretical speculation or sporadic case reports,^[8]^ lacking large-scale epidemiological data support. Confirmation of causal effects still requires RCTs of preventive interventions. Fourth, Because of the potential myelosuppressive side effects of some hypoglycemic drugs (such as certain sulfonylureas), it also seems important to exclude the effect of drugs when exploring the causal relationship between diabetes and AA, which we can improve in our future research. Fifth, our primary genetic instruments for diabetes were derived from the FinnGen biobank (Finnish population). While FinnGen provides robust genetic associations, its population-specific genetic architecture (e.g., unique allele frequencies, founder effects, and LD patterns) may limit the generalizability of our causal estimates to non-European or non-Finnish populations. Future replication in multi-ethnic cohorts is warranted to confirm these findings globally.

In conclusion, we found robust genetic evidence for a harmful association between DM and AA risk, and the immune cell trait, rCD4 + Treg AC, might have mediated this effect. The potential implications of our results for AA prevention warrant validation in well-powered RCTs. If DM truly increases AA risk via immune cells, the myelosuppression should be considered comprehensively in diabetic patients with pancytopenia. In the future, genetic engineering of Tregs may be used to treat diabetes, delay the progression of diabetes, control complications, and even promote the recovery of BM hematopoietic function. Importantly, that’s what some researchers are doing now.^[36]^

## Acknowledgments

We would like to thank the BioBank Japan, UK Biobank and FinnGen Biobank for the GWAS summary data of AA and DM. Also, we thank to the SardiNIA project which devoted to GWAS summary data of immune cell traits.

## Author contributions

**Data curation:** Chuanqi Zhong, Yuan Zeng, Tianhong Guo.

**Formal analysis:** Chuanqi Zhong, Chaoying Duan.

**Funding acquisition:** Chaoying Duan, Tianhong Guo.

**Investigation:** Yuan Zeng, Tianhong Guo.

**Methodology:** Chuanqi Zhong.

**Project administration:** Tianhong Guo.

**Software:** Chuanqi Zhong, Yuan Zeng, Tianhong Guo.

**Supervision:** Tianhong Guo.

**Validation:** Chuanqi Zhong, Chaoying Duan, Yuan Zeng.

**Writing – original draft:** Chuanqi Zhong, Tianhong Guo.

**Writing – review & editing:** Chuanqi Zhong, Chaoying Duan, Tianhong Guo.

## Supplementary Material


